# Leveraging law and ethics to promote safe and reliable AI/ML in healthcare

**DOI:** 10.3389/fnume.2022.983340

**Published:** 2022-09-27

**Authors:** Katherine Drabiak

**Affiliations:** College of Public Health, University of South Florida, Tampa, FL United States

**Keywords:** AI/ML, artificial intelligence, law, ethics, patient safety, liability, malpractice, FDA

## Abstract

Artificial intelligence and machine learning (AI/ML) is poised to disrupt the structure and delivery of healthcare, promising to optimize care clinical care delivery and information management. AI/ML offers potential benefits in healthcare, such as creating novel clinical decision support tools, pattern recognition software, and predictive modeling systems. This raises questions about how AI/ML will impact the physician-patient relationship and the practice of medicine. Effective utilization and reliance on AI/ML also requires that these technologies are safe and reliable. Potential errors could not only pose serious risks to patient safety, but also expose physicians, hospitals, and AI/ML manufacturers to liability. This review describes how the law provides a mechanism to promote safety and reliability of AI/ML systems. On the front end, the Food and Drug Administration (FDA) intends to regulate many AI/ML as medical devices, which corresponds to a set of regulatory requirements prior to product marketing and use. Post-development, a variety of mechanisms in the law provide guardrails for careful deployment into clinical practice that can also incentivize product improvement. This review provides an overview of potential areas of liability arising from AI/ML including malpractice, informed consent, corporate liability, and products liability. Finally, this review summarizes strategies to minimize risk and promote safe and reliable AI/ML.

## Introduction

Artificial intelligence and machine learning (AI/ML) is poised to disrupt the structure and delivery of healthcare, promising to optimize care clinical care delivery and information management. AI/ML offers potential benefits in medicine, such as creating novel clinical decision support tools, pattern recognition software, and natural language processing to streamline clinical encounters. AI/ML adds an additional component into the physician-patient relationship, raising questions about the appropriate role of AI/ML and ethical duties of physicians. Effective utilization and reliance on AI/ML also requires that these technologies are safe and reliable. This review describes potential flaws in the technology ranging from problems with the input data, choices that developers make during the building or training process, and how the program is eventually deployed. Potential errors could not only pose serious risks to patient safety, but also expose physicians, hospitals, and AI/ML developers and manufacturers to liability.

This review describes how the law provides a mechanism to promote safety and reliability of AI/ML systems. On the front end, the Food and Drug Administration (FDA) intends to regulate many AI/ML as medical devices, which corresponds to a set of regulatory requirements prior to product marketing and use. Post-development, a variety of mechanisms in healthcare tort law provide guardrails for careful deployment into clinical practice and incentivize product improvement. This review provides an overview of potential areas of liability arising from AI/ML including malpractice, informed consent, corporate liability, and products liability. Finally, this review summarizes strategies to minimize risk and promote safe and reliable AI/ML throughout the life cycle of development, testing, implementation, oversight, and evaluation.

## Promises of AI/ML

As of 2021, FDA approved or cleared more than 350 medical devices that use AI/ML (1). FDA states the greatest potential benefits of ML resides in its ability to create new and important insights from the vast amount of data generated during the delivery of healthcare (1). The healthcare encounter can amass data from sources including claims data, imaging, EHR documentation, genetic information, medical device sensors, and patient generated information from wearable devices (2). Medical AI/ML sifts through data curating knowledge, sorting complex interactions, and identifying patterns. The National Academy of Medicine (NAM) envisions AI/ML as a tool to advance clinical care delivery and optimize information management by offering predictions, improving performance, identifying risks, and enhancing communication (2, 3).

NAM describes three categories of use cases for AI/ML in healthcare: health monitoring systems, administration, and tools during the clinical encounter (2).

### Health monitoring systems

Health monitoring systems include wearable devices or sensors that track patient behavior and vitals. This can include heart rate sensors, sleep sensors, glucose monitors, activity trackers, and medication reminders (2, 4, 5). Wearable devices or remote monitoring could address the problem of intermittent data collection by feeding patient information directly into the EHR. This information could provide dual alerts to the clinician as well as patient facing alerts, such as progress tracking or reminders such as when to take a medication (2).

Health monitoring tools could provide insight and data on patient behavior outside the clinical setting that can better inform clinical decisions. As one example, prior to undergoing cardiac surgery a clinician could track patient weight, glucose, meals, heart rate, sleep, and activity. AI/ML may be able to provide patient specific risk assessment for operative planning, risk of complications, and post-surgical monitoring (6). Home monitoring systems can assist in additional contexts, such as for checking post-operative patients, or monitoring older adults and tracking the person's location in the home, time in bed, falls, or other aspects of patient behavior (2).

While each of these data points provides additional information about the patient with the goal of more precise care, health monitoring tools in particular also pose significant privacy concerns. Granular details about a patient's sleep, meals, activity, and location through sensors entails the most private aspects of a person's life. Moreover, some stakeholders anticipate even greater expansive data collection and connectivity across devices. NAM envisions the ability to link connected information from a patient's phone (which would include social contacts and conversations), and consumer purchasing information (such as whether the patient's report of eating certain foods matches his purchasing records) (2).

### Administrative tasks

AI/ML can streamline certain administrative tasks in healthcare management and practice. Practice managers can use AI/ML to identify peak times to modify clinical staff scheduling, optimize wait times, and identify patient no-shows (2). Natural Language Processing (NLP), a system for building a computer's ability to understand human language and transform text into readable structured data could also be useful for administrative tasks, such as clinical notetaking (7). Physicians report they spend almost half of their time working with inputting data into the EHR and other desk work such as data entry and search tasks (7). During the clinical encounter, physicians can use NLP as a tool to search and retrieve the most relevant portions of the patient record to view (8). NLP can also serve as scribe to take notes, using AI/ML as a filter to record only the most important points and directly input them into the clinical record. Finally, following the patient visit, AI/ML can assist with coding, billing, and internal automated fraud or abuse detection (2, 8, 9).

### Clinical decision support

AI/ML offer potential benefits during the clinical encounter through Clinical Decision Support systems (CDS).

CDS can interpret large amounts of data from a patient's EHR such as imaging, lab results, patient history, medication, admission history, genetic testing, and other data points (10). Physicians could oversee feeding this data into the CDS, which would compare this information along with guidelines against an intervention threshold to provide a recommendation (4). Physicians could use CDS to support a diagnosis, predict an outcome, plan treatment, prescribe and manage medications, and interpret imaging (11). CDS can sort and manage relevant clinical guidelines, providing a real time recommendation based on specific patient characteristics (12).

Many CDS systems incorporate ML, which enables computers to utilize data, learn from a dataset, and decipher patterns, recognize new correlations, and make predictions about the data without explicit programming (13). ML systems are classified as either supervised, where the system works by collecting a large number of training data that contains labeled inputs, or unsupervised, where the system uses unlabeled data to discern underlying patterns, outliers, and produce a representation of the data (6). AI/ML can also be categorized as locked or adaptive (13, 14). In locked systems, the same input will always produce the same result unless the developer modifies the program. Developers build adaptive systems, on the other hand, with the intention that they will self-update based on new data, so the input could yield a different output over time (13, 14).

CDS that integrates ML could provide potential benefits, particularly in oncology. For example, CDS could incorporate patient age, tumor size, tumor biology, and other data points the in EHR to determine the course of action for a patient with cancer (6, 10). Physicians could use CDS to select a specific type of chemotherapy, predict patient responsiveness to treatment, individualize chemotherapy dosing, or tailor radiation treatments (7, 10, 11). In an adaptive system, ML would learn from prior cases to calibrate medication dose, consider adjuvant therapies, or how to adjust radiation to minimize damage to surrounding tissue (12). Proponents of integrating CDS view the tool as a mechanism to provide precision medicine and individualized patient care that maximizes patient benefit and reduces risks (7, 10).

CDS could also improve healthcare quality and patient safety through drug management. CDS can assist physicians with selecting the appropriate type or dose of medication tailored to patient characteristics, provide reminders on drug–drug interactions, patient drug allergies, and alerts for error detection (such as wrong dosage) to reduce preventable medication errors (3, 15).

### Imaging and diagnostic medicine

Proponents of AI/ML describe useful applications in imaging and diagnostic medicine. Deep learning and ML can excel at pattern recognition and predictive modeling, which could be applied in radiology, pathology, dermatology, or during surgical procedures. AI/ML can assist by sorting and prioritizing images, screening cases, and offering clinical predictions (2, 4, 16). AI/ML can streamline practice by highlighting items a physician may miss, flag images for review, and sort through the images the system classifies as lower priority that the physician may dismiss (2, 7). In dermatology, AI/ML can screen lesions to assess whether they are benign or malignant and provide guidance for follow up testing with a dermatologist or oncologist (7, 16, 17). These same capabilities extend to applying AI/ML in other areas of oncology, such as detecting neoplastic lesions during colonoscopy, or assessing liver lesions using ultrasound imaging (16, 18). Finally, AI/ML incorporating CT or MRI can create 3D interactive anatomy models that provide information on tumor size, location, and patient vascular structure for surgical planning (19).

Some evidence suggests that AI/ML in imaging and diagnostic medicine could improve efficiency and accuracy in diagnosis, mitigate interobserver variability, and facilitate better decision-making (5, 16). Several studies suggest deep learning diagnostic systems are more effective than physicians in oncology, for example AI/ML demonstrating higher rates of correctly detecting melanoma or breast tumors as compared to specialists (17, 20). On the other hand, initial systems that appear to have high rates of accuracy may exhibit errors or lower accuracy once tested in clinical practice, which occurred with IBM Watson's partnership with Sloan Kettering. IBM initially envisioned Watson as a disruptive product to revolutionize oncology; however, the program contained multiple errors and incorrect treatment recommendations due to faulty training data (20, 21). Finally, radiologists further note that they do not simply “look at pictures,” but that their assessment involves complex decision-making, assessing numerous clinical factors with practice experience that involves more than simple detection (20).

## The relationship between AI/ML and physicians

### What's different about AI/ML?

For decades, physicians have used clinical guidelines and decision support systems based on statistical models as a tool in the practice of medicine. While early statistical models curated medical knowledge to create static clinical decision support systems, modern AI/ML builds highly complex programs involving extensive data, designed to learn from previous datasets, decipher new patterns, and relies on adaptive models that may change recommendations over time based on inputs (13). The machine's vast layers of neural networks are often referred to as a “black box,” or opaque based on the lack of transparency and explainability to outside observers (7, 20, 22). Thomas suggests this has the potential to outsource decision-making to the algorithm, which centralizes power in the algorithm and poses several concerns if physicians use the algorithm without sufficient validation and oversight (23).

Schweikart notes that AI/ML relies on diffuse development, where multiple parties such as several software and hardware developers potentially work in different locations, on different components, to create one product (22). Diffuse development without conscious coordination raises concerns about cohesion, oversight during development, and accuracy of the final product (20, 22). If AI/ML's reasoning cannot be fully understood by outsiders or explicitly stated based on complexity, this further raises questions about who is driving the decisions in healthcare and the balance of power between AI/ML and the physician.

### The role of AI/ML and the physician

Many stakeholders have addressed the issue about the appropriate interaction between AI/ML and the physician (5, 16, 20). Terry envisions different types of AI, where some tools could enhance or even substitute physician tasks as a means to improve physician performance, avoid administrative drudgery, minimize risk, and reduce time and expense (16). Some technology experts propose the concept of “enveloping,” and creating parameters of microenvironments to contain AI/ML, which preserves useful features while maintaining human control (23).

Other AI/ML developers, however, may aim to create systems that run without humans in the loop in a manner that substitutes or supersedes physician practice (22). Unlike traditional clinical guidelines or statistics-based decision support tools, AI/ML tools are distinct based on the intention for them to drive, or even replace, physicians’ independent judgment (22). Several experts caution against automating certain decisions without adequate insight about whether the recommendation is clinically optimal for the patient (4, 12, 15, 20). Froomkin et al. note that AI/ML may make decisions that are unpredictable or unclear to the physician, but correctly follow the ML algorithm (20). This constitutes a feature of AI/ML, but simultaneously raises questions about whether the physical can, or should, follow the recommendation or his own clinical judgment (20).

A closed loop system may lead to diagnostic monoculture, where clinical decisions in certain subspecialties reflect only the AI/ML recommendations, and physicians lose the ability to discover newer or better treatments (20). Overreliance on AI/ML may also result in physician deskilling, the loss of critical medical knowledge and skills, and decrease physicians’ ability to identify errors (20). While physician skill in certain areas may decrease, they would need to acquire new education and training for how to interact with new AI/ML systems (2).

### Maintaining physicians in the loop

The American Medical Association (AMA) states that AI/ML should enhance and scale human expertise rather than attempt to replace or replicate physicians (5). Notably, the practice of medicine is a distinct function specifically regulated by states laws called medical practice acts. These state laws provide precise rules for who can prescribe or administer medication, treat or diagnose disease, perform surgery, or render a medical opinion (16). Physicians, unlike machines, are also subject to legal, professional, and ethical standards of the profession such as a fiduciary obligation to the patient, truthfulness, confidentiality, and reasonable care (4). Physicians are also bound by ethical duties such as beneficence (maximizing benefit to the patient) and nonmaleficence (minimizing potential harm or risk). These ethical obligations require physicians to view the patient before them as people with distinct medical and psychosocial needs, rather than a constellation of symptoms.

The practice of medicine is also more than simply dispensing advice, but provides a critical *human* component – compassion, touch, and empathy (4, 10). As Terry aptly notes, physician engagement with each patient upholds not only ethical values underpinning medicine, but can translate to understating patient circumstances, which can also lead to improved diagnostic or treatment insights (16).

AI/ML operates without the boundaries of physicians’ ethical standards, and without the assessing the context of each recommendation. Froomkin et al. highlight that AI/ML used fixed performance criteria, but does not have the ability to self-correct to incorporate new dimensions into its value system (20, 24). Accordingly, while AI/ML may appear more efficient, consistent, or streamlines, this also comes at a cost of evaluating each specific patient's needs. The exceptional nature of the practice of medicine along with potential for error leads some stakeholders to assert that humans must remain in the loop (20, 24). AI/ML can inform clinical decision-making, but physicians must have final control to render their professional opinion in patient care.

## Potential errors and patient safety

### Potential errors when developing and deploying AI/ML

AI/ML relies on feeding data into an algorithm to produce accurate outputs, but data can be incomplete, missing, or biased (25). AI depends on data standardization, structure and organization. The sheer amount of data and fragmentation from multiple sources of records can increase risk of errors in data collection, particularly if different systems use different terminology, descriptions, and labels (26). If different healthcare institutions collect or label data differently, AI/ML trained at one institution and used at another institution may result in errors (3). If AI/ML programs use NLP to capture and record patient data directly into the EHR, the program may capture the wrong word or exclude different terminology, skewing important information in the patient's EHR.

If the EHR is connected to a continuously learning AI/ML system, this error could reverberate through the entire program (7, 25). Thomas notes that some medical data is not a recording of actual patient experience, but rather filtered through the physician's perception of the patient's state (25). Price notes the problem of contextual bias, where medical AI developed at a high resource environment such as an academic medical center may not be representative in low resource settings (7). Unrepresentative data could skew diagnostic and therapeutic patterns that AI sees, providing clinically inappropriate suggestions when applied to low resource settings (3). Nishida and Kudo also describe the potential problems of overfitting (overestimating the model's performance based on previously unencountered data) or underfitting the data (where a low capacity model is used relative to problem complexity and data size) (17). To address such potential errors, developers can work with physicians and content area experts to be involved in correctly labeling the data, select hyperparameters for input and recommendations, and tune the data (17, 25).

Current literature describes a variety of errors in AI/ML (12, 15, 27, 28). Errors can arise from a number of factors, such as software upgrades, changes to underlying datafield or code, changes in terminology, inadvertent enabling or disabling a rule, upgrading clinical information system, or database corruption (27).

Challen et al. provides an extensive description of eleven discrete quality and safety issues in AI/ML classified by whether the error occurs in the short term, medium term, or long term (12). In the short term, Challen et al. explains the potential problems of distributional shift, insensitivity to impact, black box decision-making, and unsafe failure mode (12). As one example of black box decision making, Challen et al. provides the example that an AI/ML tool that analyzes x-ray images may be inaccurate based on incorrect training data, but the opacity of the algorithm means the physician may not recognize the error, and it will not be apparent until prolonged use (12). Medium term errors include automation complacency/bias, reinforcement of outmoded practice, and self-fulfilling prediction (12). As an example of self-fulfilling prediction, a system trained on oncology outcomes may predict poor prognosis for certain patients, leading the physician to suggest palliative care rather than curative treatments, reinforcing poor prognosis outcomes (12). Finally, long-term errors include negative side effects, reward hacking, unsafe exploration, and unscalable oversight (12). As an example of negative side effects, AI/ML may learn to perform one function, such as an autonomous ventilation following surgery, but the system fails to take into account the wider context, such as inducing long term lung damage (12).

Lyell et al. classify errors in CDS as either omission or commission errors (15). Omission errors refer to when a physician fails to detect an error in the CDS, such as an incorrect treatment recommendation, prescription dose, wrong drug not indicated by clinical guidelines, or other failure to detect an anomaly. As one example, AI/ML tools that use mammography to detect breast cancer could miss lesions, or misclassify malignant lesions as benign (29). Lyell et al. classifies commission errors, on the other hand, as when physicians comply with incorrect recommendations or accept false positive alerts (15). Both errors may be driven by automation bias, or overreliance on CDS where the physician uses automated cues from the CDS to replace independent clinical assessment. The “black-box” highly complex nature of AI/ML also makes it difficult or impossible for the physician to understand the reasoning behind the CDS recommendation, creating uncertainty for a physician when comparing clinical judgment against the AI/ML recommendation as a means to prevent error (4, 15).

In addition to unintentional errors in CDS, Taitsman et al. warn against intentional bias and corruption from commercial influences (30). In 2020, the EHR vendor Practice Fusion agreed to pay $145 million to resolve criminal and civil allegations that it accepted kickbacks from fourteen pharmaceutical manufacturers in exchange for building CDS software designed to increase prescribing of manufacturers’ products (30, 31). According to the Department of Justice, in exchange for “sponsorship” payments, Practice Fusion permitted companies to influence the development and implementation of CDS alerts by setting the criteria that would trigger the alert or even draft the alert language (31). The U.S. Attorney prosecuting the case noted that CDS software in EHRs constitutes an important an important technology to inform clinical decision-making, but that prescribing decisions must be based on accurate data, reflect the patient's medical needs, and may not be tainted by “corrupt schemes and illegal kickbacks” (31).

### Impact of errors from AI/ML

In a study to assess rates of omission and commission errors in CDS, Lyell et al. tested the rates of omission and commission errors recruiting medical students to use a simulated e-prescribing system (15). Researchers informed participants that although testing found CDS alerts are highly accurate, they are “occasionally” incorrect and each participant should double check drug reference prescribing information (15). Researchers aimed to find omission errors, where the participant failed to detect a genuine prescribing error and commission errors, where the participant did not prescribe a safe medication because of a false positive alert from the CDS. Lyell et al. found when compared to scenarios with no CDS, correct CDS reduced both types of errors by 58.8% (15). However, when CDS provided participants incorrect information, this increased prescribing errors by 86.6% (15).

Notably, Lyell at al. concluded that participants had difficulty determining when CDS was wrong, but also had difficulty determining when the CDS was correct (15). CDS also can strongly influence physicians’ own judgment: Goddard et al. found evidence of commission errors from physicians changing their responses in test scenarios from correct to incorrect answers after being provided with an incorrect CDS advice (15). Translating these findings to clinical practice, physicians may have difficulty determining when CDS alerts are accurate and beneficial and should accept the recommendation, when to ignore or override the CDS, and identifying when the CDS missed a critical detail (11, 15).

Both Wright et al. and Stone describe how changes in software, hardware, lab instruments, terminology or logic in the alert builder can all contribute to errors in CDS once deployed in practice, such as failure to fire, providing an incorrect or unsafe treatment recommendation, or overfiring (27, 28). In one example, Stone describes a case study where logic in the CDS alert builder did not account for categories of drug overlap (28). In this case, a patient was already taking al alpha/beta-blocker, but the system recognized this as a separate category than a beta-blocker, and generated an alert recommendation that the physician prescribe a beta-blocker (28). Wright et al. describe a similar case, where a change in an external drug classification system caused a system-wide spike in alerts recommending redundant prescribing (27). Wright et al. also describe a case study where alerts failed to fire, such as stopping reminders to check TSH levels for patients on thyroid medications based on certain thresholds (27).

NAM notes that predictive models may help physicians asses patient risk, but methods that learn associations between inputs and outputs can be unreliable or dangerous when used to drive medical decisions without independent physician review (2).

### Accuracy in testing may not equate to clinical performance

Choudhury and Asan highlight that the accuracy of AI/ML in a model does not necessarily match the clinical efficiency or performance in practice (3). Choudhury and Asan performed a systematic literature review including 53 eligible studies that used or integrated AI/ML into clinical care (3). While initial testing may report high rates of accuracy, Choudhury and Asan assert that testing accuracy may differ from clinical performance, especially if AI/ML is tested on a small subgroup, use a small sample size, or if prediction models do not account for missing data (3). Choudhury and Asan also note that different studies use varying evaluation metrics to measure AI/ML performance (3). Some developers may report accuracy against previous versions of the same product as benchmark, rather than measuring against standard physician practice. In one example, Choudury and Asan describe how a developer advertised that its cardiovascular risk algorithm improved from one version to the next (3). However, the original algorithm that physicians used for eight years overestimated the risk of cardiovascular disease, which affected one-third of all surgeries performed during that time-period, exposing patients to potential overtreatment risks and unnecessary surgery (3).

Habli et al. note that if developers promise AI/ML safety, physicians will rely on these assurances (32). Developers’ safety assurances provides physicians confidence that the potential patient safety risks of using AI/ML are reasonably low. However, some developers’ testing systems may not account for the dynamic characteristics of the clinical setting because they cannot account for context, developers may only use limited data points, and the intended function cannot be fully represented until the system is actually deployed into clinical practice (7, 32). The adaptive nature of ML and changing clinical environments as AI/ML learns over time raise continuous questions of program confidence and patient safety (7, 32). Habli et al. assert that considerations of safety are not fully resolvable during design because certain issues may only become apparent once a healthcare institution begin to use AI/ML system (32).

### Risks to patient safety

Errors in AI/ML and CDS specifically can cause unintended adverse consequences and pose risks to patient safety (13, 28). This may include problems with the data used to develop the algorithm, choices the developers make in building or training the model, or how the AI/ML program is eventually deployed (13). Despite potential benefits, CDS are not perfectly accurate and can dispense erroneous advice. Inappropriate use can lead to the deterioration in quality of patient care such as false positives, false negatives, or add to physician workload (11). As Price aptly notes, AI/ML errors are different from other errors in medicine (7). A programming error does not merely affect a line of code, but can induce patient harm by providing unsafe, inappropriate, or missing a recommendation (25). Compared to a single error by one single physician, errors in AI/ML can potentially impact thousands of patients, resulting in mass injuries (26). Understanding the functioning of complex AI/ML requires technical knowledge that is not common among clinicians. Most physicians do not have training to identify errors and glitches, or recognize when AI/ML is providing a suboptimal or faulty recommendation (3).

Wright et al. concludes that malfunctions are widespread (27). In a survey of twenty-nine Chief Medical Officers (CMOs) on the topic of CDS errors in their healthcare institution, only two CMOs reported no CDS malfunctions (27). Thirty-eight percent of CMOs report finding CDS error four or more times per year (27). Wright et al. found users reported the error 83% of the time, and as CDS become more complex there are more areas of potential failure (27). Notably, 62% of CMOs were “not very” or “not at all” confident that existing processes in their healthcare institution were sufficient to prevent or detect CDS errors before reaching the end user (27). Importantly, Wright et al. also found that although end users knew of CDS malfunctions and risks to patient safety, software developers were mostly unaware of these outcomes and surprised by the frequency of errors (27).

Potential errors in conjunction with risk to patient safety leads several experts to caution against automation bias (4, 15, 20, 32). Haupt defines automation bias as when physicians over-rely on AI/ML, which reduces vigilance in information seeking and processing (4). If physicians over-rely on AI/ML, or CDS in particular, the physician may only provide a cursory review of the output or recommendation. In radiology, for example, if the physician relies on CDS without independent evaluation, then the physician may be more likely to overlook certain cancerous lesions on imaging tests if the AI/ML designates it as benign.

However, as Habli et al. point out, one of the central purposes of AI/ML is to delegate partial cognitive decision-making to a machine as a means to save time and improve decision-making accuracy (32). If physicians spend time developing their own opinion about the patient and whether the AI/ML recommendation aligns with their opinion and assess the potential for errors in the AI/ML, this undermines these purposes.

Haupt asserts that physicians should maintain an index of suspicion that the prediction may be wrong, and determine whether the recommendation aligns with the specific patient (4). Stone suggests that physicians should not blindly follow CDS, but consider that CDS may be missing data, the recommendation may incorporate faulty data, or lack accurate logic that could undermine its accuracy (28). Similarly, Lyell et al. recommend using CDS as an independent check for errors rather than relying on CDS as a replacement for physician judgment (15).

## Federal regulation of AI/ML products in healthcare as medical devices

### Background on medical device regulation

Federal regulations provide an important mechanism to standardize requirements for developers to demonstrate the safety and reliability of AI/ML products. NAM states that regulation should cover validation, implementation, and maintenance of AI/ML (2). FDA intends to regulate many AI/ML products as medical devices, which corresponds to a set of regulatory requirements prior to product marketing and use (14). The Federal Food Drugs and Cosmetic Act defines a medical device as: “an instrument, apparatus, implement, machine… or other similar or related article…intended for use in the diagnosis of disease or other conditions, or in the cure, mitigation, treatment, or prevention of disease” (33). FDA asserts that AI/ML has the potential to transform healthcare and produce “high value applications” such as earlier detection, more accurate diagnoses, and personalized care (14).

For medical products, FDA regulates according to a risk-based classification system, based on the device's intended use and level of risk to patients if the device is inaccurate or harmful (13, 34). Most products that are medical devices that use AI/ML are classified as software as a medical device (SaMD) (13, 14).

Class I products entail low risk, such as software that displays a glucose reading. Class II products entail moderate to high risk, such as AI/ML that sorts and classifies medical imaging findings. For most Class II devices, manufacturers must undergo a Premarket Notification 510(k) review to obtain FDA clearance prior to marketing and selling the product (35). A 510(k) review is a process where the manufacturer demonstrates the product is safe and effective for its intended use and substantially equivalent to an existing device on the market and has the same intended use and characteristics (35, 36). For other Class II devices, manufacturers can undergo a *de novo* request, which is a marketing pathway for novel medical devices where the FDA relies on general or special controls to provide reasonable assurances that the product is safe and effective for its intended use (37). Lastly, Class III medical devices are high risk products that are life supporting, life sustaining, or substantially important in maintaining human health (34). Manufacturers must undergo a full premarket review approval process and demonstrate the product's safety and effectiveness for the intended use. Once the device enters the market, FDA uses a risk-based approach to determine whether changes or updates would require additional regulatory review.

### Certain types of software are exempt from regulation

In 2016, Congress passed the 21st Century Cures Act, which amended the Food, Drug, and Cosmetic Act to exclude certain software functions from the definition of “medical device” and corresponding regulatory requirements (38). Products must meet four specific criteria:

(1) the product is not intended to acquire, process or analyze a medical image of signal;
(2) the product's purpose is only to display, analyze, or print medical information (e.g. clinical guidelines) or patient information (e.g. the patient's chart);
(3) the product can collect information about the patient and support a recommendation but does not treat the patient, make treatment recommendations, or provide a definitive diagnosis; and
(4) the healthcare provider must be able to independently review the basis of the recommendation (38).

FDA guidance provides examples that meet these criteria, such as software that displays or prints test results, displays drug labeling information, or uses the physician's diagnosis to display current treatment recommendations for a common illness such as influenza that cites the source of the clinical guidelines (38).

### FDA's framework for regulating AI/ML software

In 2019, FDA issued Guidance on Clinical Decision Support Software that applied FDA's risk based device framework using a matrix that combines the seriousness of the medical condition, the significance of information that the medical device provides, and whether developers design the product to be used by healthcare providers or patients (38).

On one side of the risk matrix, FDA provides three categories of medical conditions: non-serious, serious, and critical (38). Non-serious conditions refer to short-lived or self-limiting conditions, where an accurate diagnosis if important, but not critical to mitigate long-term irreversible public health consequences. An example of a non-serious condition would include managing mild to moderate seasonal allergies. FDA defines serious conditions as when an accurate diagnosis or treatment is of vital importance to avoid unnecessary interventions or obtain a timely intervention, such as avoiding an unnecessary biopsy. Lastly, critical conditions refer to when an accurate or timely diagnosis is necessary to avoid death, long-term disability, or serious health deterioration. Critical conditions would include the example of avoiding paralysis.

The other side of the risk matrix provides three categories to classify the significance of the information. FDA classifies the first category as CDS that is designed to inform clinical management, such as providing information on treatment, diagnosis, prevention, or aggregating relevant information. Next, driving clinical management refers to CDS designed to use in aiding treatment, diagnosis, or triage for early signs of a condition or disease. Lastly, treating or diagnosing refers to making immediate or near term action to prevent or mitigate a disease or condition, or diagnosing or detecting a disease.

Based on the intended use and function of the CDS in the risk matrix, FDA specifies whether it considers the product as a medical device and whether it intends to enforce regulatory oversight (38). In general, FDA indicated it does not intend to enforce oversight of CDS designed to inform clinical management of non-serious conditions (38). As an example of a non-serious condition, FDA states it would not enforce regulatory oversight for a ML algorithm that classifies patient specific data to provides alerts to the healthcare provider about cholesterol management. However, FDA does intend to enforce regulatory requirements for device CDS when the product is intended to inform clinical management for serious conditions or diseases and the healthcare provider cannot independently evaluate the basis of the recommendation (38). As an example, FDA describes that a ML algorithm designed to identify postoperative cardiovascular events where input and logics are not explainable to the healthcare provider would need to undergo regulatory review. As a general rule, the more serious the medical condition or disease, the greater the risk if device is inaccurate, and the less transparency of the product, the more likely the FDA will require the manufacturer to undergo regulatory review.

Additionally, FDA also stated it intends to enforce regulatory oversight of software devices that are not CDS, which may particularly impact devices used by physicians in radiology, oncology, and surgery (38). One example of software devices that still must undergo regulatory review would include products such as software that uses a patient's CT or MR imaging to create an individualized radiation treatment plan. As another example, FDA described it would also enforce oversight of software that uses a patient's CT scan to create a 3D model for surgical planning.

In contrast to the traditional regulatory process that evaluates an individual, static device, the very premise of AI/ML is leveraging patient data for continuous learning and product improvement. As of 2019, FDA had only cleared or approved AI/ML devices that relied on a locked algorithm (14). To adjust, FDA plans to offer a Software PreCertification Program (14, 39). Rather than reviewing individual devices, FDA would evaluate the developer's qualifications, processes to produce safe and effective devices, and whether the developer can demonstrate compliance with Good Machine Learning Practices (14, 39). Good Machine Learning Practices include adhering to best industry best practices for algorithm design, training, and testing (14). Prior to submission, developers would describe the Predetermined Change Control Plan, which would outline plans for future device modifications with specific protocol for changing the algorithm (14). Developers would submit the Predetermined Change Control Plan with the device's initial regulatory application and describe processes relating to data management, data retention, software performance evaluations, update procedures, and ongoing performance monitoring. If developers make modifications within the bounds of the Predetermined Change Control Plan, they would document the changes and submit notice to the FDA. However, if modifications lead to a new use (such as expanding to a new target population), this would require undergoing a full review process.

### FDA regulation moving forward

Following FDA's Guidance on Clinical Decision Support Software, several stakeholders including the AMA responded with specific concerns (40). AMA noted FDA's sole focus on potential benefits of AI/ML and minimization of potential risks (40). AMA expressed concern that FDA presumes collecting and using more data constitutes an inherent good and perpetuates the viewpoint that AI/ML merely reflects a neutral and objective mathematical process. According to the AMA, identifying variation or flaws in datasets is critical for assessing impact to patient safety. AMA suggested that FDA should connect developers’ goals for the AI/ML to measurable patient outcomes to increase the transparency and assess the impact of AI/ML.

In 2021, FDA published Software as a Medical Device Action Plan, which builds upon principles from the 2019 guidance and outlined additional plans to increase transparency to users and patients, address algorithmic bias, and assess real world performance (39).

## Liability for AI/ML

Regulatory standards provide a minimum threshold to ensure products in the marketplace are safe and effective for the intended use. Despite regulatory clearance or approval, medical devices can still contain flaws, errors, defects, and cause patient harm. Potential errors in AI/ML products could not only pose serious risks to patient safety, but also expose physicians, hospitals, and AI/ML manufacturers to liability. Tort liability for AI/ML encompasses potential claims such as malpractice, breach of informed consent, corporate negligence, and products liability (10, 18, 20, 22). The tort law system serves several important functions, including deterrence (reducing unsafe products or medical practices), incentivizing optimal standards (increasing quality products and attentive patient care), and compensation for injuries.

### Malpractice

Physicians have a duty to uphold the standard of care when interacting and treating patients. If physicians deviate from the standard of care, and this departure from the standard of care is the cause of an injury to the patient, the physician may be held liable. Schweikart describes several potential scenarios that could give rise to malpractice liability (22). First, AI/ML could recommend a course of action, but the physician believes another course of action is more prudent based on professional experience (22). If the physician overrides the AI/ML and the patient is injured, the patient may assert that the AI/ML recommendation constitutes the standard of care and the physician's deviation from that standard was the cause of his injury. Second, AI/ML may recommend a course of treatment, the physician may follow the recommendation and the patient is injured (22). The patient may assert that the physician should not have followed the AI/ML because it was wrong, and the physician's decision to rely on the AI/ML is what caused the patient's injury.

Froomkin et al. note that the opacity of AI/ML poses potential difficulties in the context of potential malpractice claims (20). The lack of interpretability for neural networks makes it difficult to pinpoint a specific source of error, if any, in the ML based prediction system. This creates uncertainties of deciphering whether the AI/ML made an error which the physician should not have relied upon, or whether the physician should have followed the AI/ML recommendation. The “black box” nature means there is no practical way for physicians to understand the reasoning or articulate the decision-making process of the machine (20). This creates a new duty for physicians to critically evaluate AI/ML they use in practice, and potential liability for relying on faulty AI/ML (18).

### Informed consent

During a clinical encounter, physicians have a legal and ethical obligation to inform patients of material information pertinent to a treatment plan. Physicians have a duty to disclose information about benefits, risks, and alternatives relating to a proposed course of treatment (22). U.S. jurisdictions reflect a split on how to determine what constitutes material information. Some jurisdictions rely on the “reasonable physician” standard, which states that physicians have a duty to disclose information that a reasonable physician under the same or similar circumstances would disclose (6). Other jurisdictions, however, apply the “reasonable patient standard,” which examines whether a reasonable patient would attach significance to the risk and want the physician to disclose that specific risk (6).

Several legal and ethics scholars have discussed whether the doctrine of informed consent would require physicians to disclose to patients when they are using or relying upon AI/ML during the clinical encounter to make a diagnosis or recommend a treatment (4, 6, 22, 41). Schweikart describes potential problems relating to physicians using AI/ML and informed consent (22). If AI/ML presents treatment plans in absolutes without pros and cons, this creates difficulty for understanding the source of the recommendation, verifying its accuracy, and how physicians should communicate this to patients (22).

Cohen points out that the amount of required disclosure is closely tied to perception of the technology (6). Those who believe that adopting AI/ML entails potential risks or proceeds too quickly would favor requiring the physician to disclose to the patient if he uses or relies on AI/ML. From the opposite perspective, those who believe AI/ML offers complex but unassailable benefits favor nondisclosure.

Cohen adopts the position that physicians would not have a duty to disclose when they use or rely upon AI/ML (6). Cohen asserts that under the reasonable physician standard, physicians would likely consider AI/ML's recommendation as one step of many along the decision-making process (6). Physicians do not regularly disclose the minutiae of every step of their decision-making to patients. In fact, according to Cohen, too much disclosure is costly, inundates patients with complex and confusing information, undermines patient's distinguish meaningful risks from trivial risks, and may lead patients to distrust physician recommendations based on AI/ML (6). Similarly, under the reasonable patient standard, Cohen asserts that patients would not find a physician's reliance on AI/ML material, and patients only want a warrant of the AI/ML's credibility, which would be satisfied through regulatory oversight (6).

Haupt and Findley et al., on the other hand, suggests that physicians may have a duty to disclose when they use or rely upon AI/ML (4, 41). This article suggests that under the reasonable physician standard, physicians have a duty of transparency when providing a diagnosis or treatment recommendation. In this context, transparency equates to the physician explaining in direct and simple terms the basis for a decision, such as an imaging scan, genetic test, or other diagnostic test. If physicians use AI/ML as another decision-making tool, it may be reasonable that physicians have a duty to disclose this to patients. Applying the reasonable patient standard, some suggest that reasonable patients would find a physician's use of AI/ML constitutes a material fact that the physician has a duty to disclose. Froomkin et al. note that patients already have a negative reaction to AI/ML if a physician discloses this during the patient encounter (20). Similarly, Findley et al. report that patients show an aversion or bias against algorithmic decisions (41). Some research demonstrates that people prefer human physicians to AI/ML for diagnosis, screening, and treatment (41). Other research finds that some people accept AI/ML in diagnosis and planning, but are less accepting of other technologies such as partially autonomous surgeries (41). Under the reasonable patient standard, this article suggests that if AI/ML supplements or replaces a physician's independent judgment with an opaque or unexplainable algorithm, this may in fact be material to the patient and triggers a duty for disclosure.

### Corporate liability

Hospitals, healthcare systems, or physician groups could also face liability from physician errors and injuries related to malpractice. The doctrine of vicarious liability recognizes that employers may be liable for acts committed by their employees if the employees are acting within the scope of their employment based on the theory that employers exercise a form of supervision and control over their employees. If a patient alleges a malpractice action against a physician relating to using AI/ML, the patient may also bring suit against the physician's employer under the theory of vicarious liability.

Healthcare institutions could also face liability for corporate negligence relating to using or relying on AI/ML in the facility. Healthcare institutions have four non-delegable duties that they owe directly to patients: (1) a duty to use reasonable care in the maintenance of safe and adequate facilities and equipment; (2) a duty to select and retain competent physicians; (3) a duty to oversee all persons who practice medicine within the institution; and (4) a duty to formulate, adopt, and enforce adequate rules and policies to ensure quality care for their patients (42).

Under the first duty, institutions must ensure equipment is working property and maintain a minimum level of safety. As applied to AI/ML, this would correspond to confirming potential AI/ML tools such as NLP programs connected to patient EHRs, software imaging tools, and CDS operate (and continue to operate) in an adequately safe manner without error. Second, if the institution embeds certain AI/ML features (for example, all clinical encounters use NLP for recording EHR notes), then the institution may have a duty to ensure physicians’ competence in interacting with such software by requiring minimum training or education on AI/ML. Third, institutions’ duty to oversee physician practice corresponds to credentialing physicians, monitoring patient outcomes, flagging anomalies in physician performance, and addressing instances where physician actions increase risk of adverse patient outcomes. Institutions may have a duty to credential certain physicians with adequate training to use certain types of AI/ML. Institutions should also assess whether physicians using certain types of AI/ML improves patient outcomes and monitor for potential errors that could pose risks to patient safety. Finally, institutions would need to adopt rules and policies related to selecting, deploying, and monitoring AI/ML tied to patient safety metrics.

### Products liability

Patients who assert the AI/ML was the cause of an injury may also bring suit against the manufacturer of the AI/ML. Products liability permits people to seek recovery and compensation when they are injured by products that are not reasonably safe due to a design detect, manufacturing defect, or the manufacturer's failure to warn (10, 20). Griffin provides an extensive description of each type of products liability claim and potential examples in the context of AI/ML (10).

Under a design defect claim, a product is defective in design when (1) foreseeable risks of harm posed by the product (2) could have been avoided or reduced by the adoption of a reasonable alternative design and (3) the omission of the alternative design renders the product not reasonably safe (10, 20). To succeed in this claim, the plaintiff must demonstrate all of these three elements (10). First, the plaintiff would need to show that the type of injury he suffered is foreseeable, such as demonstrating how poor data can cause flaws in a ML algorithm. Second, the plaintiff would show the manufacturers could have used a reasonable alternative design, such as a device without AI/ML, a different dataset, or a more user friendly interface. Lastly, jurisdictions differ on the standard they apply for what constitutes a product that is not reasonably safe. Here, the question would focus on whether the AI/ML performed as well as another AI/ML system or met the performance specifications provided by the manufacturer. Griffin provides useful examples when a plaintiff may allege the product is not reasonable safe, such as if the physician does not have adequate space to fully document patient symptoms, or the patient received a delayed diagnosis because the AI/ML relied on outdated imaging.

In manufacturing defect claims, the plaintiff must demonstrate (1) product was defective, (2) that the product caused the plaintiff's injury, and (3) the defect existed at the time the product left the manufacturer's control (10). On example of this type of claim in AI and robotics involves *Taylor v. Intuitive Surgical* (10, 43). In this case, a patient who underwent a prostatectomy by a physician using the da Vinci robot alleged that the robot was defective when it left the manufacturer. The plaintiff alleged the robot had microcracking that caused electricity to escape during the procedure that caused internal burning to the patient's rectum, resulting in a variety of post-surgical complications including infection, renal failure, respiratory failure, incontinence, and reliance on a colostomy bag (10, 43). Though the trial court rule granted a ruling in favor for Intuitive Surgical on this specific claim, it raises an important consideration for other AI/ML manufacturers (43).

Manufacturers may also have a duty to warn institutions that purchase the products and physicians that use the products of potential risk. A manufacturer may be liable if a plaintiff can show the product is defective because of (1) inadequate instructions or warning (2) when the foreseeable risks of harm posed by the product could have been reduced or avoided by the provision of reasonable instructions or warnings by the seller and (3) the omission of the warnings renders the product not reasonably safe. In at least one jurisdiction, manufacturers have a duty to warn hospitals that purchase the device in addition to warning the physician that uses the device (43). The variety of potential errors in AI/ML combined with Wright et al.'s conclusion that CDS malfunctions are widespread suggests that manufacturers may have a duty to warn institutions and physicians against using AI/ML as the sole basis of diagnosis or treatment, but instead one of several decision-making considerations (10, 26, 43).

Anticipating potential areas of liability can incentivize physicians to critically evaluate their reliance on AI/ML, promote institutional responsibility when implementing new AI/ML into a hospitals and healthcare systems, and encourage manufacturers to create more carefully designed products.

## Steps to address accountability, explainability, and reliability for AI/ML

Current literature provides a number of strategies to anticipate and address issues of safety, accountability, and reliability of AI/ML (3, 11, 17, 28, 32, 44). Developers and healthcare institutions can use these strategies in conjunction with FDA regulations to minimize legal risk, address potential ethical concerns related to adopting AI/ML, and guide the development of best practices.

Habli et al. state that the potential for errors raises questions about safety assurances and moral accountability for potential harm to patients (32). Physicians do not exercise control over decisions or recommendations that the AI/ML makes, and based on the opaqueness of AI/ML physicians’ ability to understand how the system translates the data is difficult or impossible (32). Some experts suggest that the lack of explainability reduces the transparency of decision-making and undermines the ability for physicians to remain accountable when dispensing diagnostic and treatment recommendations (32, 44). Froomkin et al. note that although most algorithms have high traceability (running the same program will achieve the same result), they also have low explainability (they cannot provide a short narrative of why the program arrived at this reasoning) (20).

Physicians’ moral accountability in professional judgment is important because it deters professional complacency, underpins patient trust in medical care, and fosters goodwill from patients (32, 45). Zawati and Lang describe how accountability and trust are integral components to the healthcare system (45). Trust is closely aligned with the concept of informed consent, and the patient's belief that the physician's recommendation furthers the patient's best interests. Public trust is also connected to patients’ willingness to follow treatment recommendations, or even seek treatment in the first place. If patients perceive AI/ML has uncertain accountability or ambiguity of who is really in control of the recommendation, patient trust in the healthcare system and physicians may erode (45). Habli et al. asserts that moral accountability acts as a mechanism to drive decision-making that aligns with the patient's best interest, but this creates a problem if AI/ML instead drives clinical decision-making (32).

Mahadevaiah et al. outline five steps to minimize risk, prioritize patient safety, and maximize user acceptance (11). Char et al. provide complementary ethical considerations mapped to steps of the AI/ML process, including during development, implementation, evaluation, and oversight (44).

### Product development

Char et al. begins with suggestions for developers, asserting that developers should consider the reasons and transparency behind algorithm design, training data, training process, and validation data (44). Here, transparency standards should identify whether the AI/ML is locked or adaptive, and designed to be assistive or autonomous. Char et al. assert that non-inspectable black box systems that lack explainability pose risks to patient safety and are subject to catastrophic failures (44). To remedy this during development, manufacturers should create systems that are transparent and auditable, with an “explainable architecture,” where developers outline the decision-making process in a manner that is familiar to physicians, aligns with human cognitive decision-making processes, and is tied to clinical evidence (44).

### Selection

Mahadevaiah et al. describes the process of selection, whereby a team of interdisciplinary clinicians and health professionals consider potentially adopting AI/ML (11). Mahadevaiah et al. assert that implementing AI/ML such as CDS should be part of a wider, coherent quality improvement strategy (11). Char et al. suggest that the healthcare institution should address the question of why it is selecting this clinical area, articulate the desired outcome, and define the end goal in relation to the AI/ML (44). Clinicians and health professionals should assess areas where there is a clinical quality gap relating to processes or patient outcomes, and ensure data exists to suggest that implementing CDS would reduce the quality gap. These assessments, according to Mahadevaiah et al,. should compare performance metrics such as time saved, diagnostic accuracy, health outcomes, or process improvement from physician practice alone to physicians using CDS (11).

Teams of technology experts working at the implementation site such as the hospital can audit and review the proposed algorithm design, training data, training process, validation methods, and initial outcomes prior to clinical implementation (44). While the AI/ML may still pose explainability barriers to physicians, this interprofessional approach provides an additional layer of review to mitigate against conflicts of interest or error. Many experts recognize that by design, the AI/ML will not be fully explainable to physician users (7, 20, 22, 25). Developers and healthcare institutions should be able, at the minimum, to provide an alternate checklist to physicians: how the AI/ML was developed, potential risks of using it, limitations, and whether it is appropriate for the physician's patient population (22).

During the selection process, healthcare institutions should consider the content of information the AI/ML is designed to provide to the physician. The healthcare institution should ensure that the information is clinically relevant, brief, and unambiguous (11). The strength of the evidence and behind the recommendation should be apparent to the user. The AMA suggests adopting a labeling system to alert users to relevant information such as regulatory status (such as whether FDA has approved or cleared the device), and percent representations of safety and efficiency (40). Healthcare institutions should also consider the usability of the AI/ML and the amount of training required to operate the system.

### Validation, performance and calibration

In this stage, the healthcare institution should verify developers’ claims about completeness, data quality, and effectiveness. Several authors caution that developers may overstate product benefits and that institutions should create independent testing methods using unenriched data to test CDS performance in each institution's system (3, 11).

Developers should work with healthcare institutions to test the clinical completeness, comprehensiveness, consistency, and repeatability of AI/ML in different settings (11, 17, 44). Validation may include measures of sensitivity, specificity, and positive predictive value (17). In the healthcare setting, sensitivity refers to accuracy for providing true positive results, while specificity refers to correctly generating a negative result (44). A higher rate of sensitivity translates to fewer missed diagnoses and missing treatment opportunities, while a higher rate of specificity equates to fewer false positives that could correspond to inappropriate and harmful overtreatment.

Tomaszewski and Gillies describe validating the connection between biological correlates, the disease process, and clinical outcomes to the AI/ML output (46). In radiomics specifically, Tomaszewski and Gilles suggest biological validation can provide a critical connection between the result of radiomic analysis and the clinical decision process (46).

Nishida and Kudo recommend calibrating the AI/ML and measuring how effectively the predicted probability matches the actual diagnosis (17). This is the stage where developers and healthcare institutions would work to identify the acceptable level of variability, for example in assessing the same lesion based on different images and work to adjust parameter settings (17). Char et al. assert that to maximize benefit and minimize harm, AI/ML must perform in accordance with cardinal design features of safety, efficiency, and equity (44). Safety requires AI/ML with features that prevent injuries and hazards. For example, this could include prospective planning to address typical error scenarios such as unexpected, incorrect, or incomplete data, abrupt closure (such as a power outage or system failure while using AI/ML), and error messages (11). Efficiency refers to AI/ML that solves the specific problem developers designed it to address at reasonable costs, including the costs of false negative or false positive diagnoses (44). Finally, equity refers to sharing the advantages of AI/ML in a fair manner (44).

Mahadevaiah et al. outline several steps for pilot testing CDS on real world cases in clinical practice (11). Mahadevaiah et al. suggests testing CDS in parallel to the existing workflow to assess clinical relevance, user acceptance, physician adherence to CDS recommendations, impact on patient decisions, and clinical outcomes (11). During this stage, Mahadevaiah et al. describe customizing CDS to assess and improve the appropriateness of alerts to avoid alert fatigue (11). Stone states that the alert itself should provide context and background for the recommendation, which will increase transparency and enhance the physician's ability to accurately judge the appropriateness as applied to the patient (28). To anticipate and address potential errors, a steering committee consisting of developers/technology experts, clinicians, and administrators can identify relevant issues, difficult cases, or rare situations where CDS may fail (11, 28). Mahadevaiah et al. assert that testing and improvements during this phase will build future physician confidence and acceptance (11).

### Implementation, evaluation, and oversight

Implementation entails designing and executing the rollout plan, transitioning from an old workflow, and adopting a new process that incorporates AI/ML in the healthcare institution (11). Several experts recommend new education initiatives in medical education and continuing education to prepare physicians to evaluate and interpret AI/ML systems (3, 26).

In this phase, healthcare institutions should continue to assess whether characteristics of AI/ML change in real world applications and perform ongoing safety evaluations (44). Some safety and quality issues, according to Habli et al. are not fully resolvable during design stages and only become apparent once the healthcare system deploys the product (32). Quality assurance systems should assess performance and safety of AI/ML by measuring performance with set metrics on efficiency and efficacy, which could include measuring the sensitivity and specificity of AI/ML, change in patient health outcomes, or resources saved such as productivity or costs. Systems should build in mechanisms to receive and act upon user feedback, such as rate of alert firing, errors, and anomaly detection so the healthcare system can work to identify and remediate problems in real time (11, 28). Mahadevaiah et al. suggest tracking and monitoring when physicians followed or declined a CDS recommendation, stating that this can offer insights to modify the program or identify an undetected malfunction (11).

Over time, health care institutions should monitor AI/ML performance and quality to measure, mitigate, and correct for external context drift and internal model drift. External context drift refers to when patterns of clinical practice change over time, changes in patient case mixture, or the obsolescence of certain treatments of drugs (11). This can lead to shift in the AI/ML where AI/ML such as CDS makes recommendations that no longer align with the most recent or best clinical guidelines (11). Internal model drift refers to including new datasets, new cutoff values, or parameters for recommendations and designing updates to reflect changes in the models underpinning AI/ML (11). Finally, Mahadevaiah et al. recognize that adaptive AI/ML and real time updates offer the benefit of continuous improvement, but simultaneously raise the risk of undetected degradation from bias in input (11). Importantly, Habli et al. assert that safety assurances should not be static, but rather dynamic throughout the lifecycle of AI/ML (32).

## Discussion

AI/ML tools such as CDS, pattern recognition software, and NLP harness the potential to transform healthcare. Capitalizing on data aggregation, AI/ML could provide useful alerts; offer precise models to guide medication, radiation, and surgical plans; increase personalized treatment recommendations; and reduce physicians’ administrative burden. AI/ML offers the promise of enhancing, complementing, and streamlining the practice of medicine. Despite potential benefits, AI/ML carries risks of potential errors during data collection, development, and deployment. Unlike a single physician error, errors in AI/ML impacts patients system-wide, and by design may be less visible due to the opacity of AI/ML. Reducing risk of errors and promoting reliable AI/ML is critical to protecting patient safety.

This review provides useful points to consider for each of the following stakeholders: (1) physicians/clinicians; (2) AI/ML developers and manufacturers; and (3) hospitals and healthcare institutions.

AI/ML has the potential to disrupt the physician-patient relationship, by scaling and augmenting – not replacing – physician capabilities. The practice of medicine is governed by state law and bound by ethical obligations that recognize medicine constitutes more than mechanized advice, but a human discipline of healing and compassion.

Physicians will likely require additional training to become proficient in using new AI/ML systems, but should remain cautious of automation bias, deskilling, or abdicating judgment to the system. While correct AI/ML can reduce errors, incorrect recommendations are both difficult to spot and pose significant risks to patient safety. Accordingly, physicians should maintain an index of suspicion when interpreting an AI/ML recommendation and use AI/ML as an additional piece of information or assistive tool rather than a replacement for clinical expertise.

In the event that patients do suffer an injury arising from a physician using AI/ML, physicians may face potential liability for malpractice or lack of informed consent. Using AI/ML creates a new obligation for physicians to critically evaluate the AI/ML they use in practice, and potentially disclose to patients when an AI/ML recommendation forms the basis of a physician's diagnosis or treatment recommendation. Federal regulations classify many types of AI/ML as medical devices, which provides a series of requirements designed for developers and manufacturers to demonstrate evidence that software is safe and effective for its intended purpose. Developers and manufacturers should be aware that FDA's new regulatory model places a greater burden on developers to demonstrate safety and effectiveness of the software over the lifecycle of the product, including accounting for algorithm changes, updates in hardware and software, or shifts in clinical practice.

Developers should work closely with healthcare institutions in the process of creating new AI/ML that is driven by clinical need or part of a quality improvement strategy. Developers should be aware that accuracy in testing does not equate to accuracy in clinical performance, and that certain safety issues require testing, validation and calibration in a healthcare institution. Importantly, current literature demonstrates a disconnect between developers’ perception of product accuracy and the actual occurrence of errors and malfunctions reported by physicians and health administrators. This suggests that developers should integrate features to provide ongoing product feedback to ensure developers are alerted to (and can expediently correct) product errors.

In the event that patients do suffer an injury arising from a physician using AI/ML, this also raises potential liability against the developer or manufacturer. Liability provides a mechanism of accountability for developers to carefully consider how to minimize foreseeable risks of using AI/ML, such as sufficient testing, validation, and design safety. Manufacturers may also have a duty to warn institutions or physicians of the potential risks of using the product, including potentially providing a label warning against using the AI/ML as the sole basis of diagnosis or treatment.

Hospitals and healthcare institutions have an obligation to carefully assess new technologies prior to adoption and integration. Health administrators and corporate executives should be aware that regulatory approval or clearance provides a minimum standard when determining the safety and efficacy of AI/ML, but that AI/ML may still contain errors, defects, and flaws resulting in risks to patient safety.

Before selecting AI/ML to use in the institution, the institution should address why it is selecting the product as part of a quality improvement strategy, how the product will improve clinical outcomes, or physician experience. The institution should clarify what constitutes the desired outcome metrics (time saved, diagnostic accuracy, health outcomes, process improvement), and what data exists to support the proposition that this product will lead to this objective. Institutions will need to work closely with developers during the validation, performance and calibration stages to test real world performance and assess impact to workflow, user acceptance, and actual patient outcomes.

Institutions have a legal duty to ensure they provide safe equipment, adequately train and oversee physicians in the institution, and enact policies and procedures to ensure quality patient care. In the context of AI/ML, this places a burden on the institution to assess the benefits and risks before adopting new products, ensure physicians have adequate education for the uses and limitations of AI/ML products (including credentialing processes for using AI/ML), and develop procedures for feedback mechanisms.
